# Automatic Classification of Sub-Techniques in Classical Cross-Country Skiing Using a Machine Learning Algorithm on Micro-Sensor Data

**DOI:** 10.3390/s18010075

**Published:** 2017-12-28

**Authors:** Ole Marius Hoel Rindal, Trine M. Seeberg, Johannes Tjønnås, Pål Haugnes, Øyvind Sandbakk

**Affiliations:** 1 Centre for Elite Sports Research, Department of Neuromedicine and Movement Science, Faculty of Medicine and Health Science, Norwegian University of Science and Technology, 7491 Trondheim, Norway; pal.haugnes@ntnu.no (P.H.); oyvind.sandbakk@ntnu.no (Ø.S.); 2 SINTEF DIGTAL, P.O. Box 124 Blindern, NO-0314 Oslo, Norway; Trine.Seeberg@sintef.no (T.M.S.); Johannes.Tjonnas@sintef.no (J.T.)

**Keywords:** accelerometer, gyroscope, IMU, inertial measurement unit, neural network

## Abstract

The automatic classification of sub-techniques in classical cross-country skiing provides unique possibilities for analyzing the biomechanical aspects of outdoor skiing. This is currently possible due to the miniaturization and flexibility of wearable inertial measurement units (IMUs) that allow researchers to bring the laboratory to the field. In this study, we aimed to optimize the accuracy of the automatic classification of classical cross-country skiing sub-techniques by using two IMUs attached to the skier’s arm and chest together with a machine learning algorithm. The novelty of our approach is the reliable detection of individual cycles using a gyroscope on the skier’s arm, while a neural network machine learning algorithm robustly classifies each cycle to a sub-technique using sensor data from an accelerometer on the chest. In this study, 24 datasets from 10 different participants were separated into the categories training-, validation- and test-data. Overall, we achieved a classification accuracy of 93.9% on the test-data. Furthermore, we illustrate how an accurate classification of sub-techniques can be combined with data from standard sports equipment including position, altitude, speed and heart rate measuring systems. Combining this information has the potential to provide novel insight into physiological and biomechanical aspects valuable to coaches, athletes and researchers.

## 1. Introduction

Cross-country skiing is a whole-body exercise endurance sport wherein skiers use skis and poles to propel themselves forward across hilly terrain on snow. The two main styles employed in cross-country skiing, classic and skating, consist of several sub-techniques that skiers frequently make transitions between due to changes in speed, incline, or snow conditions. These sub-techniques are performed as cyclical movements, with the exception of the tuck (TCK) position used in downhill sections. The main sub-techniques in classical cross-country skiing are diagonal stride (DIA), double poling with kick (DK) and double poling (DP) [1]. In addition, the herringbone technique (HRB) [2], where the skis are angled and edged to increase static friction, is used in steep uphill terrain, the TCK position is used for downhill portions and various turn (TRN) techniques are employed at narrow turns [3].

In recent years, micro-sensors, specifically inertial measurement units (IMUs) consisting of an accelerometer, a magnetometer and a gyroscope, have been attached to athletes for subsequent analysis of sub-techniques and various cycle characteristics while skiing [4,5]. Several studies have aimed to automatically classify the sub-techniques used in both classical and skating style cross-country skiing, including studies on snow and roller skiing. For example, roller ski skating sub-techniques were classified using a machine learning method, a Markov chain of multivariate Gaussian distributions, and data from an IMU in a smart phone strapped to the skier’s chest [6]. While that study reported an accuracy of 98%, the analyses were based on a limited dataset of only a total of 438 cycles. The authors also reported that the extracted sequence used for classification went through a manual visual inspection. Their work was later extended to a larger study with skiing on snow in [7], where analyses of 11 skiers reached an average accuracy of 86% ± 8.9%. However, by allowing the algorithm to be developed based on data from the individual skiers in the training data, resulting in a less generalized algorithm, the average accuracy rose to 90.3% ± 4.1%.

In a follow-up study, a single IMU attached on the skier’s upper back was used to classify classical cross-country skiing sub-techniques using an automatic algorithm based on numerous hard decision rules [8]. That study reported an accuracy of 83.8% in correctly identified cycles in DIA, DK, DP, TCK, TRN and a miscellaneous class that identified cycles that did not fit in their other categories. Here, HRB was defined as DIA. The same algorithm was used in a recent study [9], reporting that the cycles in “93.3% ± 2.0% of the race distance could be identified correctly”. However, this was achieved after the data were “examined visually for errors in classification”, meaning that the accuracy achieved was not from a fully automated algorithm. This indicates that the accuracy achieved by a human well-trained to detect cycles, referred to as the human baseline accuracy, of classifying classical sub-techniques from IMU data is approximately 93%.

There are several studies where data from multiple IMUs on arms and legs/skis have been used to automatically identify sub-techniques using hard decision rules for both skating [10] and classical cross-country skiing [11]. An accuracy of 94.8% was reported when using data from roller ski skating [10] and 98.5% when roller skiing in the classical style [11]. However, as pointed out by Marsland et al. [9] “roller skiing tends not to be performed on courses with technical (sharp) corners”, which are the parts where it is most difficult to classify sub-techniques.

We have earlier classified classical sub-techniques on snow using hard decision rules on data from IMUs on arms and legs [12], resulting in classification sensitivity and precision of 99% to 100% for DIA, DK and DP. However, only three main classes were classified with the rest of the data classified as “unknown”. Additionally, the algorithm was dependent on data from four sensors that can be expensive and impractical to attach/detach with frequent use. In the current literature, it seems to be a trend that more sensors are included in order to achieve improved classification accuracy. However, more sensors lead to systems that are inconvenient for the athletes to use in their daily training and races.

In the present study, we aimed to reduce the number of sensors and improve the overall accuracy of the automated classification of classical cross-country skiing sub-techniques by using two IMUs attached to the skier’s arm and chest together with employing a statistical machine learning algorithm, commonly referred to as a neural network [13]. Specifically, we introduced a robust cycle detection for classical cross-country skiing by using data from a gyroscope placed on the skier’s arm wrist to subdivide the dataset into cycles, and we use accelerometer sensor data from an IMU on the chest to classify each cycle into the different sub-techniques. The choice of using only two sensors is to reduce the sensitivity of data loss and to make it more convenient to wear, for example, by integrating one IMU with the heart rate sensor on the chest and the other in a sports watch on the arm. Compared to the previous methodologies [6,7,8,9,11,12], we have refined the division of classes by adding HRB, TCK and TRN as separate classes and introduced the classifications of transitions to DIA (tDIA) and from DIA (fDIA). Our hypothesis was that a machine learning algorithm with a robust cycle detection and a refinement of the sub-techniques could classify sub-techniques with significantly higher accuracy than in previously reported studies.

## 2. Materials and Methods 

### 2.1. Sensor Data

The sensor data were recorded using a previously described multi-sensor system [12] that can synchronize data from seven IMUs with an accuracy of at least 30 ms. However, in this study only data from two of the IMUs were used: the accelerometer data of the chest sensor and the gyroscope data from one arm. The sensor data were sampled at 20 Hz.

**Figure 1:**
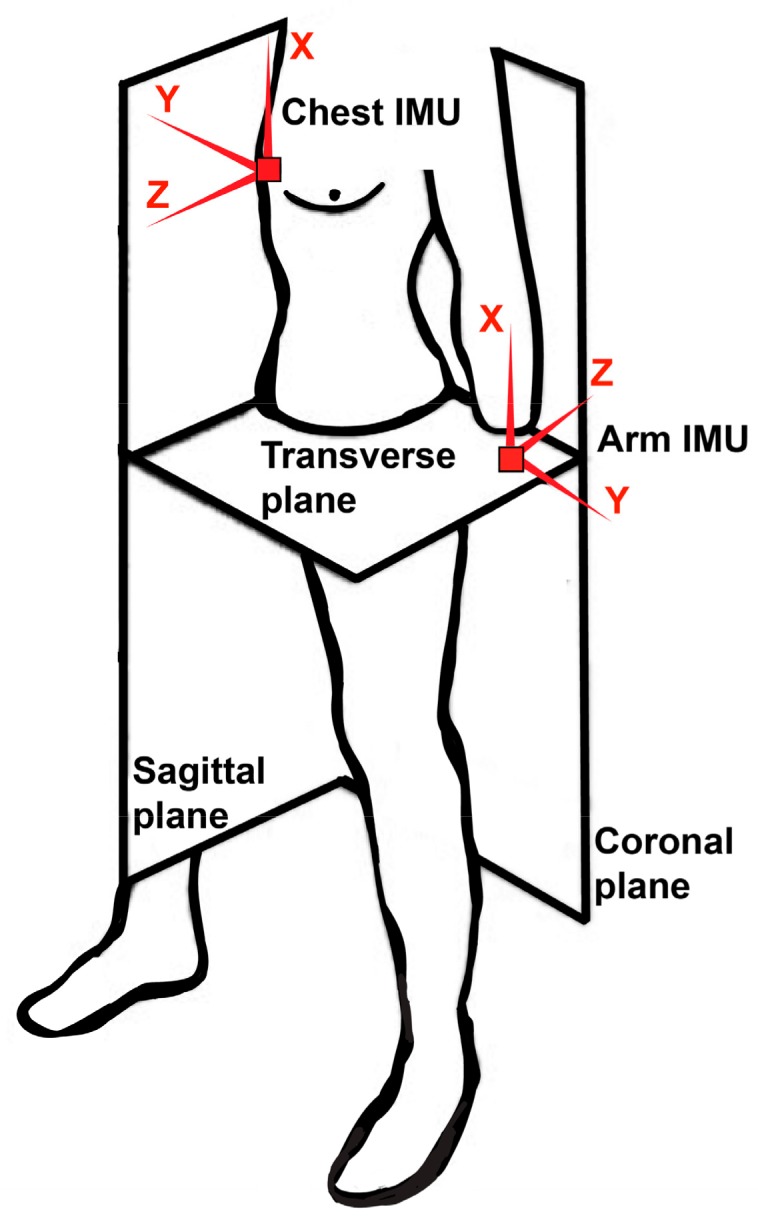
Illustration of the definition of the sagittal, coronal and transverse anatomical planes relative to the athlete. The figure also indicates the sensor locations with the definition of the corresponding x, y and z-axis for the chest sensor and arm sensor.

The sagittal, coronal and transverse anatomical planes are defined in Figure 1. With the sensor placed on the chest, our definition of the positive x-axis is perpendicular to the transverse plane, the positive y-axis is perpendicular to the sagittal plane, and the positive z-axis is perpendicular to the coronal plane. As the athlete moves, the coordinate system of the sensor will rotate within the defined anatomical planes.

### 2.2. Cycle Detection

A natural way to classify a dataset of cross-country skiing into sub-techniques is to divide the dataset into individual cycles and then classify each cycle. This approach was, to the authors’ knowledge, first utilized in [6] using a single IMU on the skier’s chest to detect and classify movement cycles when doing skating-style skiing. However, cycle detection using a single IMU on the skier’s chest for both cycle detection and classification is more challenging in classical cross-country skiing. There is a large difference in the movement pattern for the chest while performing the different sub-techniques. During DIA, the chest is relatively still and the movement is mostly performed by the arms and legs, while for DK and DP the whole upper body rotates in the sagittal plane.

**Figure 2:**
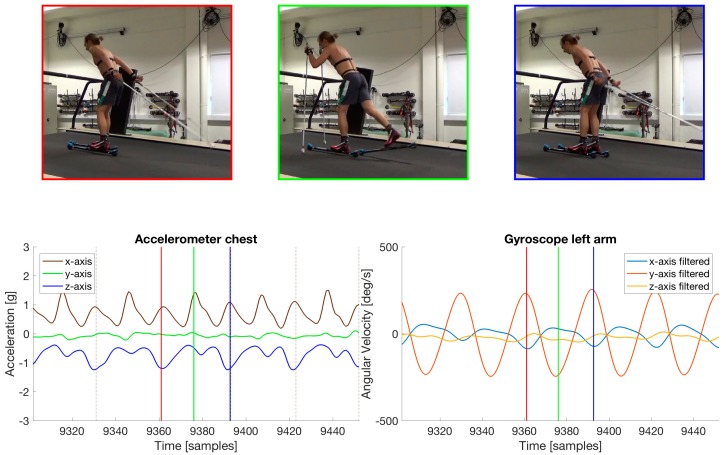
The cycle detection is done by filtering the gyroscope data from one of the arms. The y-axis results in a signal with a clear sinusoidal shape. By synchronizing this signal to a video of the skier we can observe that the peaks in the signal correspond to when the arm is extended all the way behind the athlete. This is indicated with the red and blue vertical lines in the plots. The red and blue vertical lines correspond to the video-frame displayed above with the corresponding color. The minima of the sinusoid on the y-axis corresponds to when the arms plant the poles in front of the skier, as illustrated by the green vertical line and film frame.

In order to get a robust cycle detection, we have introduced the use of a gyroscope on the arm of the skier. The signal from the y-axis has been used for cycle detection. The IMU-sensor on the arm has the coordinate system as indicated in Figure 1. To compensate for any misalignments in the mounting of the arm sensor, the coordinate system is calibrated based on the assumption that the axis with the most angular motion is perpendicular to the sagittal plane [12]. The calibrated gyroscope data were then filtered with a Gaussian low-pass filter, with 0.25 s (5 samples) standard deviation in the time domain. The hard filtering of the y-axis results in a signal with a clear sinusoidal output (see the lower right plot in Figure 2). Figure 2 also shows how this signal has been synchronized to a video of the skier. The peaks in the y-axis gyroscope signal corresponds to the movement when the arm is extended all the way behind the athlete. The red and blue vertical lines correspond to the video-frame displayed above with the corresponding color. The minima of the sinusoid on the y-axis corresponds to when the arms plant the poles in front of the skier, as illustrated by the green vertical line and the film frame in the same figure. This is true for all sub-techniques. We define a cycle as starting and ending when the arm is extended all the way behind the athlete. In the lower left plot of the accelerometer data from the chest in Figure 2, all the cycles detected are indicated with brown vertical dashed lines. Thus, by detecting the peaks of the filtered gyroscope data, we get a robust cycle detection. For sub-classes such as TCK, where the arms do not move much, the cycles detected will be much longer in length/time since the movement of the arm is insufficient to create a detectable peak in the gyroscope data from the arm. This can also be the case for some TRN cycles where the athlete does not move the arms during the turn, but most turn movements involve sufficient movement of the arms to detect the individual cycles.

### 2.3. Definition of Classes

**Figure 3:**
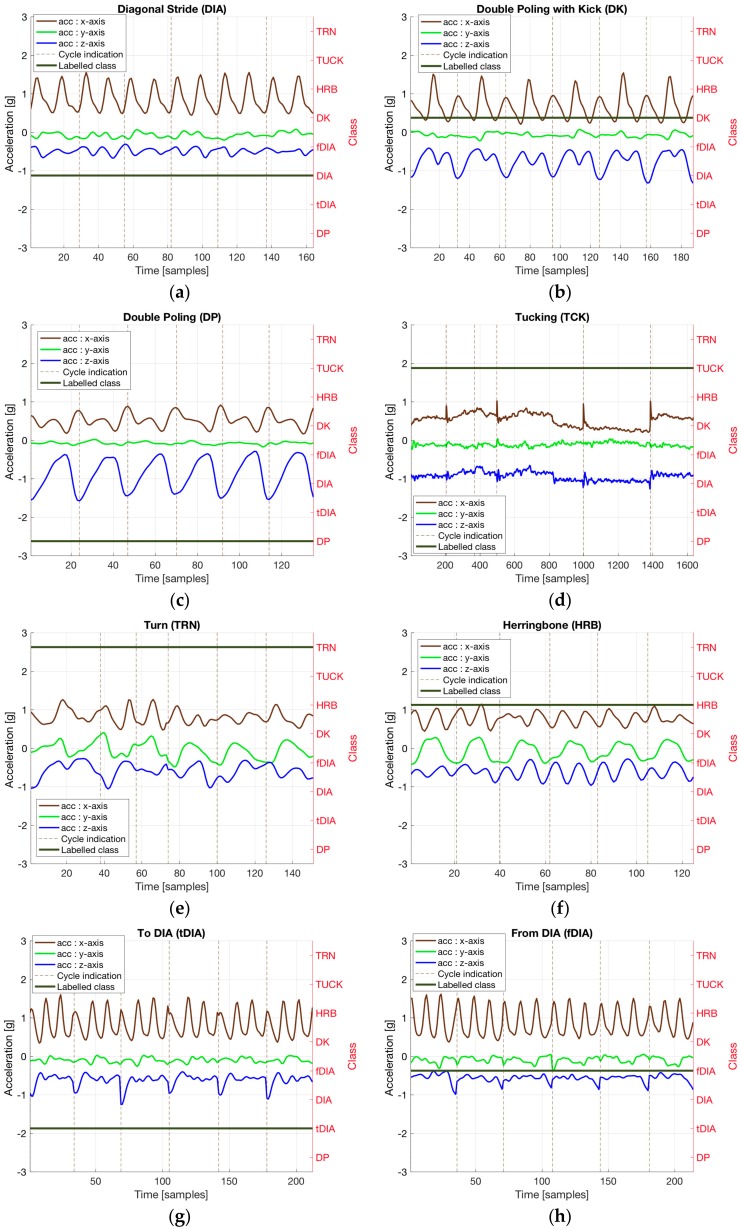
Six cycles of each sub-technique class definition are plotted in (**a**) for DIA, (**b**) for DK, (**c**) for DP, (**d**) for TCK, (**e**) for TRN, (**f**) for HBN, (**g**) for tDIA, and (**h**) for fDIA.

In Figure 3, six cycles of each sub-technique are plotted. In addition to the well-defined sub-techniques DIA (Figure 3a), DK (Figure 3b), DP (Figure 3c), TCK (Figure 3d), TRN (Figure 3e) and HRB (Figure 3f), two more classes have been defined: when the skier is changing to DIA (tDIA, Figure 3g) from DP or DK, one of the arms has to be held back to get the non-synchronized diagonal movement of the arms. The same, but in the opposite direction, is true when changing from DIA (fDIA, Figure 3h) to DK or DP since one of the arms has to be held back in order to obtain the synchronous movement of the arms. Therefore, some of the cycles are slightly different from the regular DIA when changing to and from DIA, often with an extra kick visible as an extra spike in the x-axis.

Visually there are differences between all sub-techniques. For example, for DIA (Figure 3a) each kick is seen on the x-axis, while for DP (Figure 3c) there are no kicks, but a larger cyclic movement in the z-axis, indicating the rotation of the chest in the sagittal plane. DK is a merge between DP and DIA with one kick for every cycle while the cyclical movement of the chest is maintained. TCK has a relatively constant speed, meaning little acceleration on all axes, thus the flat response in Figure 3d. HBN is similar to DIA, but there is a clear sideways acceleration (in the coronal plane) visible on the y-axis in Figure 3h. TRN is more irregular, but we can see large sideways acceleration (in the coronal plane) on the y-axis in Figure 3e, which is expected in a turn movement. Although tDIA and fDIA are both very similar to DIA (see Figure 3g,h), these cycles often have an extra kick, and the rising and falling edge of the z-component shows that the skier is either raising the chest from a DP or DK cycle or lowering the chest into a DP or DK cycle.

### 2.4. Participants

In total 10 different subjects were part of this study, 9 males and 1 female, age 30 ± 7.6 years (range 16–43), body height 180 ± 6 cm, body mass 73.0 ± 8.4 kg. The athletes ranged in skills from professional world-cup skiers to trained amateurs (competing in recreational races), and the tracks included were indoor roller skiing on a treadmill in a lab (Granåsen, Trondheim, Norway), outdoor cross-country skiing on snow in competitive tracks (Holmenkollen and Natrudstilen), and recreational tracks (Sjusjøen and Sognefjellet). The testing protocols and procedures were explained verbally to the participants and written informed consent was obtained. The Norwegian Centre for Research Data (NSD, Bergen, Norway) approved the study.

### 2.5. Defining the Feature Vectors

The feature vectors describing each cycle were created by interpolating or decimating the samples from the three axes of the accelerometer data from the IMU sensor on the skier’s chest. The coordinate system for the chest sensor is indicated in Figure 1. While we calibrated the coordinate system to compensate for misalignment in the mounting of the arm sensor, this was not necessary for the chest sensor since the mounting of the chest sensor was more robust. The accelerometer data were filtered with a Gaussian low-pass filter, with a 0.0875 s (1.75 samples) standard deviation in the time domain, to remove high frequency noise and movements not contributing to the separation of sub-techniques. The samples were decimated or interpolated into 30 samples per cycle and then appended into one feature vector of 90 samples. Additionally, four more values were added: the original length of the cycle in samples and the normalized sum of the samples from each axis. This gives the feature vector a total length of 94.

### 2.6. Training the Neural Network

The neural network was implemented using the Neural Network Toolbox in MATLAB 2016a (The MathWorks, Natick, MA, USA). A total of three hidden layers with respectively 50, 10 and 20 neurons in each layer were used, and the Bayesian regularization backpropagation was used as the training scheme [14]. Datasets from six different athletes, five males and one female, were used to train the neural network. The datasets used for training are summarized in Table 1. All the datasets used for training combined resulted in a total training dataset consisting of 4308 training cycles that were manually labeled according to class.

In this study, either the left or the right arm has been used for cycle detection. This was done to compensate for partly missing data in the dataset. For most of the sub-techniques, whether the movements within one cycle was symmetrical is independent of the use of the left or the right arm for defining the beginning and end of a cycle. An exception is the HBN sub-technique. The sideways movement, acceleration in the y-axis, is dependent on which arm defines the beginning and end of the cycle.

To make the algorithm robust against cycles defined with the left or right arm, we created an artificially larger training dataset by taking every cycle in the original training data and created a new cycle by keeping the x-axis and z-axis, whereas the y-axis was flipped. This way, we got a total training dataset of 8616 cycles. The neural network was trained 20 times to account for the randomness of the training process. A separate validation dataset, not a part of the training data, was used to evaluate which of the 20 trained neural networks we should finally use. The neural network with best performance in terms of accuracy on the validation dataset was selected.

### 2.7. GNSS Trajectory with Sub-Technique Indication

The IMU sensor data were synchronized with heart rate and GNSS data from a Garmin Forerunner 920XT (Garmin Ltd., Olathe, KS, USA) sports watch, using both GPS/GLONASS and a barometric altitude monitor, to measure position and altitude with a sampling frequency of 1 Hz. The sub-technique classification can be visualized as GNSS trajectories, with different colors indicating the sub-techniques or by visualizing the sub-techniques used in different terrain sections with information about altitude, incline, speed and heart rate.

### 2.8. Statistical Analysis 

The statistical analysis includes conventional measurements of accuracy such as the mean accuracy with a standard deviation when the accuracy of multiple datasets is combined. For a more in-depth analysis of the classification results, the confusion matrices [15] of the classified results are presented. The confusion matrix is used as it gives a detailed presentation of the classification model performance, distinguishing between: wrong classification X of true Y and a wrong classification Y of a true X. The concept is further explained in the next section based on the results.

## 3. Results

### 3.1. Training Data Results

**Figure 4:**
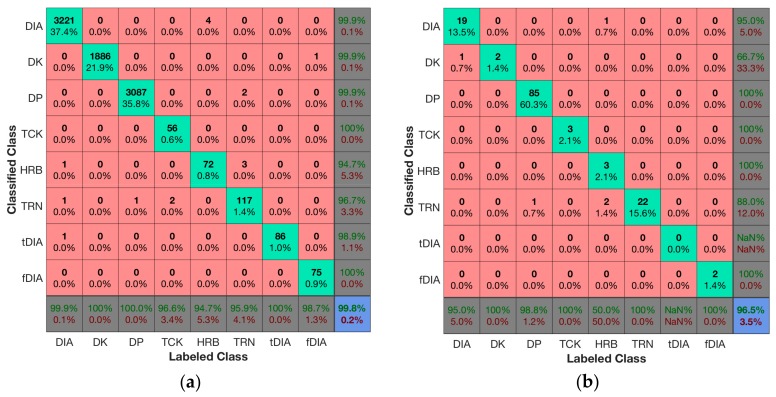
Confusion matrices for the training data (**a**) and validation data (**b**) showing that the overall accuracy for the training data (**a**) was 99.8%, although it dropped to 96.5% for the validation dataset. The accuracy can be read from the lower right element of the matrix, while the bottom row reports the sensitivity for each class, and the right most column reports the precision. The diagonal shows the true positive values for each class (labeled and classified as that class), while the numbers off the diagonal report any confusion between the classes.

The neural network, trained according to Section 2.6, resulted in a 99.8% accuracy on the training dataset and 96.5% accuracy on the validation dataset. The confusion matrix for the training data is presented in Figure 4a and the confusion matrix for the validation dataset in Figure 4b. The concept of the confusion matrix is explained while referring to the confusion matrix of the training data plotted in Figure 4a. The columns represent the labeled class and the rows the classified class. The point where the row and column for a certain class intersects indicates the true positive values for that class. The first element in the upper left quadrant of the matrix in Figure 4a therefore specifies that the training set had 3221 cycles correctly classified as DIA. In the fifth column of the first row there is a value of 4—meaning that four cycles labeled as HRB were classified as DIA. In the fifth row of the first column, we find the number 1, indicating that one cycle was labeled as DIA but classified as HRB. These two observations tell us that there is some confusion between the classes DIA and HRB in the training dataset. The numbers in the percentage reported in the bottom row of the matrix are the sensitivity (true positive rate) for each class, while the percentage in the right most column represents the precision (positive prediction value). We can, for example, observe that the class with the lowest sensitivity in the training data is the HRB technique with a 94.7% sensitivity. This is because of the four cycles that were labeled as HRB but classified as DIA. There were 72 cycles correctly classified, resulting in a sensitivity of $\frac{true\;positive}{true\;positive+false\;negative}=\;\frac{72}{72+4}=0.947=94.7\%$. HRB also has the lowest precision, while TRN has the second lowest precision of $\frac{true\;positive}{true\;positive+false\;positive}=\;\frac{117}{117+4}=0.967=96.7\%$, since two cycles were labeled as TCK, one labeled as DIA and one as DP while being classified as TRN. The lower right element of the matrix reports the overall accuracy, which we can read is 99.8% for the training dataset.

### 3.2. Test Data Results

To assess the performance of the suggested algorithm, ten new test sets from six different subjects, including the female subject, were used. None of these test sets were part of the training data, and four of the subjects never had any recorded datasets used as training data. To create the test sets, the sensor data were synchronized to a video of the athlete, and a cross-country skiing expert labeled each detected cycle based on the video as one of the eight classes. All cycles, except cycles that were not properly defined because of lost sensor data from the arm or chest sensor were labeled. The cycles not labeled were excluded (EXC) from the classification. These test sets give the accuracy of the classifier, and also test how well it adapts to new datasets from subjects that have never been a part of the training datasets.

The overall results from all the test sets are summarized in Table 2. The table shows the subject, the location of where the dataset was recorded and the numbers of cycles classified as each sub-technique class versus the numbers of cycles labeled to that class. The EXC column is the numbers of cycles excluded from the classification of the dataset because of missing sensor data. Additionally, the last column shows the accuracy. The mean dataset accuracy was 94% ± 3%.

**Figure 5:**
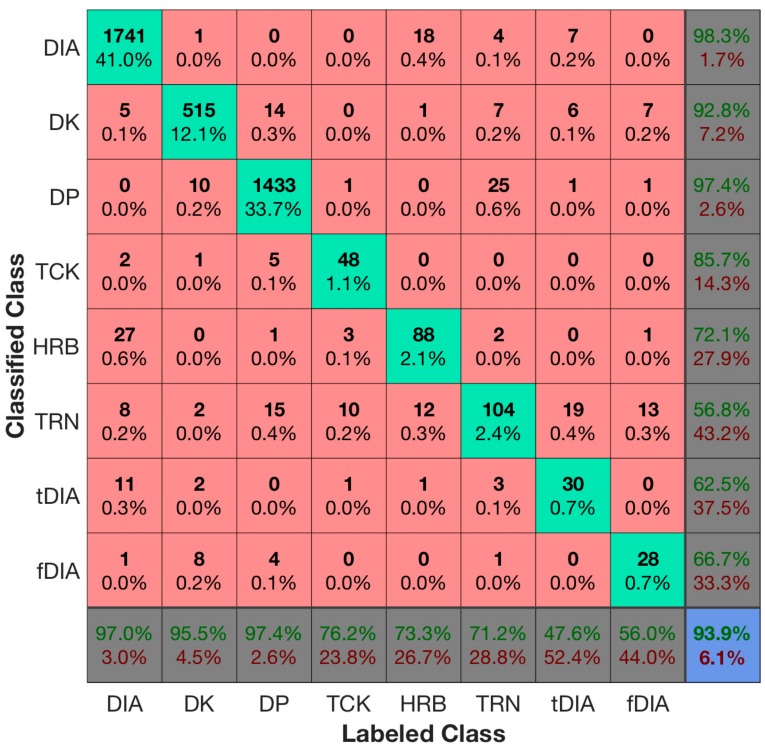
A combined confusion matrix for the all the test datasets. We can, for example, observe that the overall accuracy was 93.9% (lower right value), DP had the best sensitivity (lower row) of 97.4% and DIA had the best precision (rightmost column) of 98.3%. Also, fDIA had the worst sensitivity of 47.6%, and TRN had the worst precision of 56.8%.

A confusion matrix of all the test sets combined into one dataset are shown in Figure 5. From the confusion matrix, the main classes DIA, DK, DP all have sensitivity and precision in the 90-percentile. The other classes have lower sensitivity and precision. However, since the classes with low precision and sensitivity occur much more seldom than the main classes, the overall accuracy of the combined test sets are 93.9%.

In Appendix A, the entire test set from subject 1 skiing at Natrudstilen and from subject 7 at Sognefjellet have been plotted. These two datasets were selected because they had the best and worst accuracy, respectively, not including the test set from the laboratory, which, as expected, had the best accuracy since lab conditions are much easier than conditions in the field.

### 3.3. GNSS Trajectory with Subclasses-Indicated

**Figure 6:**
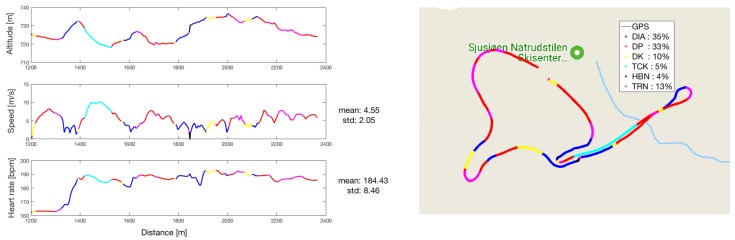
The dataset from subject 1 at Natrudstilen plotted as a GNSS trajectory (to the right) with subclasses indicated by color. The plots to the left display the height (altitude), speed and heart rate with sub-classes indicated by color.

**Figure A2:**
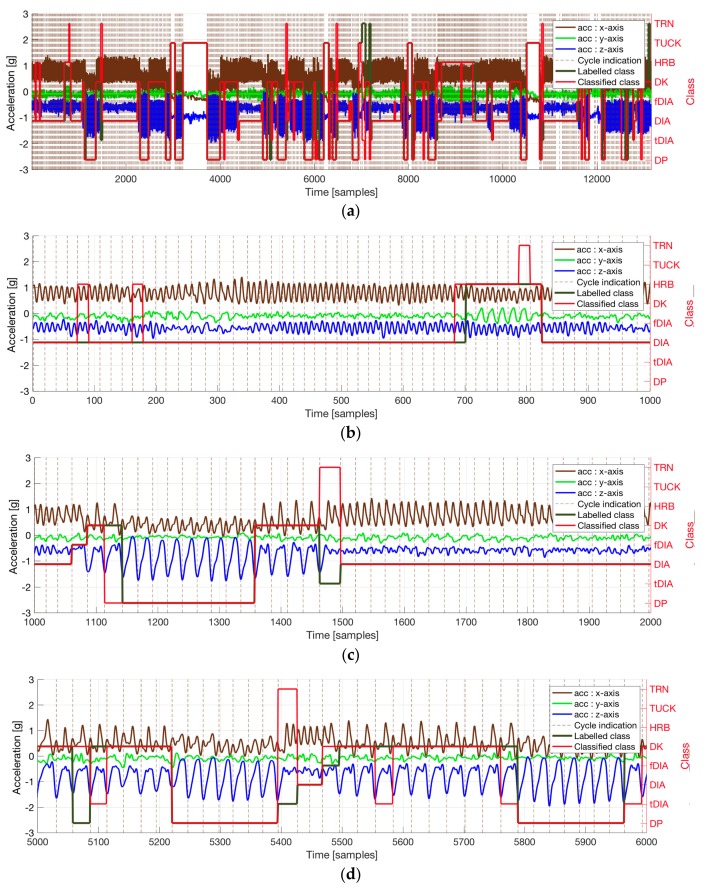

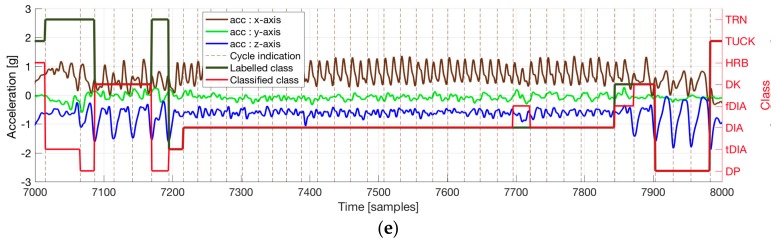
The entire test dataset from subject 7 at Sognefjellet is plotted in (**a**), while (**b**–**e**) show an enlarged view of the parts of the dataset where most misclassifications occur.

The classified sub-techniques are plotted as a GNSS trajectory together with the estimated height and speed in Figure 6 for subject 1 skiing at Natrudstilen, providing more insight into the dataset. As expected, the sub-technique TCK was used when the speed was highest, which occurs during steep downhill. The opposite was observed for DIA, which is usually used at lower speeds in uphill terrain. The same was true for HRB, which is used at especially low speeds in the steepest parts of the course. We can also observe that it is the DIA sub-technique, used in uphill terrain, that raises the heart rate the most. From the trajectory, we can observe that the TRN sub-technique was used in the parts of the course with high curvature. Appendix A, Figure A2 shows the same observations for subject 7 at Sognefjellet.

## 4. Discussion

The presented algorithm, using a neural network to classify sub-techniques in cross-country skiing, reached an average accuracy of 94%, which is slightly better than the reported accuracy reached when automatic classification was manually corrected in Marsland et al. [9]. Hence, our methodology is probably approaching the highest possible accuracy for the classification of sub-techniques in classical style cross-country skiing. While the previous methods often report that the incorrect classification occurred “especially during transitions between different gears” [7], our algorithm approached this problem by introducing a robust cycle definition using a separate IMU on the skier’s arm. Additionally, we solved the problem of misclassification by including the detection of transitions to and from DIA as separate classes. By separating these transitions into specific classes, we also avoid confusing the main DIA class with training data from the transitions cycles, which, as we saw in Section 2.3, have slightly different sensor data. This was also the reason why we decided to separate HRB into a separate class, which is different from previously reported algorithms and introduces the possibility of analyzing where the athlete switches from DIA to HRB and vice versa.

A weakness of our approach is that some of the classes, such as HRB, tDIA and fDIA, have low classification sensitivity and precision. In the confusion matrices in Figure 4a,b, the overall results of the training and validation data were fairly good, but for the test data in Figure 5 the tDIA specificity is only 47%. This results from 18 cycles being labeled as tDIA that was classified as TRN as well as some being wrongly classified as DIA, DK and DP. An overall observation is that the classes with the lowest amount of training data also have the lowest classification rates. This was also expected since more training data results in better generalization and improved classification accuracy [13]. HRB, tDIA and fDIA seldom occur in the datasets, whereas there are moderate amounts of TCK and TRN. We expect the classification accuracy of these specialized classes to increase as we acquire more training data. However, since the specialized classes seldom occur in the datasets, the overall accuracy is improved with the introduction of the specialized classes as compared to the methods previously published in the literature. One could also merge DIA, tDIA and fDIA and HRB into one class in a post-processing step to avoid the confusion between these classes since it often is sufficient to know in which sections DIA was used.

An interesting aspect related to the classification accuracy is to what extent we can trust the labels from the expert. For example, defining if a cycle is HBN or DIA can be challenging since the defining boundary between HBN and DIA is a grey area. It is not always trivial to define if the skis are angled so much that the cycle should be HBN and not DIA, especially since elite skiers aim to keep the angles as small as possible at high speeds [2]. The same discussion applies to the difference between TRN and DP, where the angle between the skis and the amount of lateral work are differentiating factors. Hence, there will always be some inconsistency and uncertainty in the labeling of the dataset and thus some insecurity in the accuracy reported. We therefore believe that accuracies in the upper 90th percentile are the highest possible when including all cycles from a skiing course. Another challenge for an automatic algorithm is that there will always be cycles not fitting the model or pre-defined classes. For example, these can be cycles where the skier stretches the back, arms and legs, or looks at the watch. When such cycles are included, as we have done in this study, a perfect accuracy is probably not achievable since the athletes may always perform unexpected movements not fitting the algorithm or model defined.

Nevertheless, the accuracy achieved in the current study is sufficient to undertake automatic classification of sub-techniques confidently. In Figure 6, sub-techniques are aligned with the GNSS trajectory and combined with altitude, speed and heart rate, illustrating how sub-technique classification can be used to provide useful analyses of a cross-country skiing competition or training. Additionally, this methodology allows unique scientific analyses of integrated physiological and biomechanical aspects in outdoor skiing. Although future studies should investigate this in greater detail, the current study shows some interesting trends worth mentioning. For example, the sub-techniques used seem highly related to skiing speed, both since faster skiers use other sub-techniques and since speed is related to incline. At the highest speed, skiers use TCK and at the lowest speed HBN. We also observe that heart rate rises most in the DIA sub-technique. DIA is used in parts of the course with steep inclines where muscles in both the upper and lower extremities are used extensively. Future studies should also include cycle kinematics, such as cycle lengths and cycle rates, as well as velocity thresholds for switching between sub-techniques, as recently conducted by Marsland et al. [9]. With the current approach, such analyses can be done automatically and reduce the time consumed for manual corrections as previously performed [9].

## 5. Conclusions

The current study presents a novel approach using machine learning on data from two IMUs for automatic classification of classical cross-country skiing sub-techniques, reaching the highest overall accuracy ever reported in the literature when reporting from larger dataset from skiing on snow. This is done by utilizing a robust cycle detection from an IMU on the arm of the skier and by defining specific classes for cycles where skiers transition between sub-techniques. However, the overall accuracy can possibly be further improved if more training data on the specific classes are acquired.

The classification of sub-techniques in classical cross-country skiing has reached the accuracy necessary to confidently provide novel insight into physiological and biomechanical parameters when combined with data from standard sports equipment such as position, altitude, speed and heart rate.

## Figures and Tables

**Table 1:** Table summarizing the datasets from the six subjects used to train the neural network and details of the validation dataset. Each row represents one separate dataset, and the columns indicate how many cycles belong to each class.

Dataset	DIA	DK	DP	TCK	HBN	TRN	tDIA	fDIA
Training Data:								
Sub. 1 Granåsen/lab	266	79	433	0	0	0	0	0
Sub. 1 Holmenkollen 1	255	85	201	0	0	0	0	4
Sub. 1 Holmenkollen 2	0	0	22	0	0	0	0	0
Sub. 1 Holmenkollen 3	0	50	0	0	0	0	0	0
Sub. 1 Holmenkollen 4	70	0	0	0	0	0	0	0
Sub. 1 Natrudstilen 1	80	22	54	4	10	13	3	3
Sub. 1 Natrudstilen 2	82	15	59	6	7	8	3	4
Sub. 1 Natrudstilen 3	48	1	54	1	11	11	0	2
Sub. 3 Natrudstilen 1	29	7	39	2	10	15	0	0
Sub. 4 Natrudstilen	33	15	46	4	0	14	2	0
Sub. 5 Sjusjøen	256	176	124	12	0	0	17	14
Sub. 9 Natrudstilen	394	432	428	0	0	0	18	11
Sub. 10 Natrudstilen	96	61	84	0	0	0	0	0
Percentage of dataset	37.4%	21.9%	35.8%	0.7%	0.9%	1.4%	1%	0.9%
**Validation dataset:**								
Sub 3. Natrudstilen 2	20	2	86	3	5	22	2	0
Percentage of dataset	14%	2%	61%	2%	4%	16%	1%	0%

**Table 2:** Table summarizing the classification results of the 10 test sets from 6 subjects. Each row represents one test dataset while the columns indicate the sub-techniques. The numbers above the slash is the number of cycles classified as the class indicated in the column header, while the numbers below the slash is the number of cycles labeled as the class indicated in the column header.

Dataset	DIA	DK	DP	TCK	HBN	TRN	tDIA	fDIA	EXC	Acccuracy
Sub. 1 Natrudstilen	83/80	18/16	72/74	1/1	10/14	18/16	3/3	3/4	0	96%
Sub. 1 Sognefjellet	198/194	92/79	149/154	4/5	3/6	9/17	5/8	6/3	5	93%
Sub. 2 Sognefjellet	202/205	64/61	129/137	2/3	8/6	35/19	3/10	2/4	50	92%
Sub. 2 Granåsen/lab	263/265	74/75	479/482	5/0	0/0	0/0	2/1	0/0	0	99%
Sub. 5 Natrudstilen	98/103	18/16	43/42	6/5	4/1	20/21	4/4	3/4	0	94%
Sub. 5 Sognefjellet	249/267	39/37	203/206	10/18	21/21	38/12	13/7	3/8	3	91%
Sub. 6 Sognefjellet	165/165	55/53	151/144	6/7	6/5	26/28	0/8	5/4	55	92%
Sub. 7 Sognefjellet	203/205	105/114	107/97	10/12	49/47	6/8	9/8	11/9	0	91%
Sub. 7 Sognefjellet	210/218	70/70	72/70	7/7	13/5	8/7	9/10	8/10	4	95%
Sub. 8 Sognefjellet	100/93	20/18	66/66	5/5	8/15	23/18	0/4	1/4	0	92%

**Figure A1:**
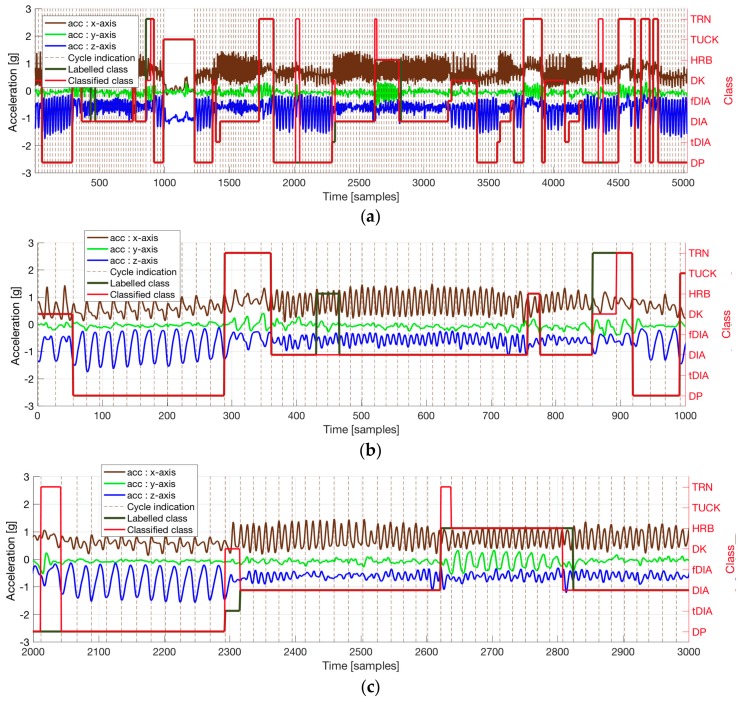
The entire test dataset from subject 1 at Natrudstilen is plotted in (**a**), while (**b**,**c**) shows an enlarged view of the parts of the dataset where the misclassifications occur.

**Figure A3:**
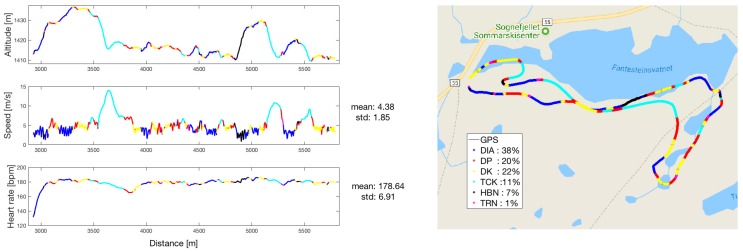
The dataset from subject 7 at Sognefjellet, the same as plotted in Figure A2, plotted as a GNSS trajectory (to the right) with subclasses indicated by color. The plots to the left display the height (altitude), speed and heart rate with sub-classes indicated by color.

## Appendix A

Figure A1 shows the entire test set from subject 1 skiing at Natrudstilen, and Figure A2 shows the entire test set from subject 7 at Sognefjellet. These two datasets were selected since they had the best and worst accuracy, respectively, not including the test set from the lab.

### Appendix A.1. Test Set from Subject 1 at Natrudstilen

Figure A1a shows the entire dataset from subject 1 at Natrudstilen, while Figure A1b,c show an enlarged view of the parts of the dataset where the misclassifications occur. We can observe in Figure A1b that there are two cycles at about sample 430 that were labeled as HRB but were classified as DIA and one cycle at sample 880 labeled as TRN but classified as DK.

In Figure A1c, one cycle at sample 2010 was labeled as DP but classified as TRN, one cycle at sample 2300 was labeled as tDIA but classified as DK, one cycle at 2620 was labeled as HRB but classified as TRN and one cycle at 28,100 labeled as HRN but classified as DIA.

### Appendix A.2. Test Set from Subject 7 at Sognefjellet

Figure A2a shows the entire dataset from subject 7 at Sognefjellet, while Figure A2b–e show an enlarged view of the parts of the dataset where most of the misclassifications occurred. The overall interpretation of the results is that there is some confusion between DIA and HBN, there is also a tendency for irregular cycles to be classified as TRN. Most misclassifications occur in the transition between sub-techniques.

### Appendix A.3. GNSS Trajectory with Subclasses-Indicated for Subject 7 at Sognefjellet

The classified sub-techniques are plotted as a GNSS trajectory together with the estimated height and speed in Figure A3 for subject 7 skiing at Sognefjellet, providing more insight into the dataset. See Section 3.3 for comparison.
